# Tumor Heterogeneity in Glioblastomas: From Light Microscopy to Molecular Pathology

**DOI:** 10.3390/cancers13040761

**Published:** 2021-02-12

**Authors:** Aline P. Becker, Blake E. Sells, S. Jaharul Haque, Arnab Chakravarti

**Affiliations:** 1 Comprehensive Cancer Center, Ohio State University, Columbus, OH 43210, USA; Saikh.Haque@osumc.edu (S.J.H.); Arnab.Chakravarti@osumc.edu (A.C.); 2 St. Louis School of Medicine, St. Louis, MO 63310, USA; blake.sells@wustl.edu

**Keywords:** glioblastoma, tumor microenvironment, precision medicine, glioma, neoplastic stem cells, prognosis, tumor heterogeneity, review

## Abstract

**Simple Summary:**

Glioblastomas (GBMs) are the most frequent and aggressive malignant tumors arising in the human brain. One of the main reasons for GBM aggressiveness is its diverse cellular composition, comprised by differentiated tumor cells, tumor stem cells, cells from the blood vessels, and inflammatory cells, which simultaneously affect multiple cellular functions involved in cancer development. “Tumor Heterogeneity” usually encompasses both inter-tumor heterogeneity, differences observed at population level; and intra-tumor heterogeneity, differences among cells within individual tumors, which directly affect outcomes and response to treatment. In this review, we briefly describe the evolution of GBM classification yielded from inter-tumor heterogeneity studies and discuss how the technological development allows for the characterization of intra-tumor heterogeneity, beginning with differences based on histopathological features of GBM until the molecular alterations in DNA, RNA, and proteins observed at individual cells.

**Abstract:**

One of the main reasons for the aggressive behavior of glioblastoma (GBM) is its intrinsic intra-tumor heterogeneity, characterized by the presence of clonal and subclonal differentiated tumor cell populations, glioma stem cells, and components of the tumor microenvironment, which affect multiple hallmark cellular functions in cancer. “Tumor Heterogeneity” usually encompasses both *inter-tumor heterogeneity* (population-level differences); and *intra-tumor heterogeneity* (differences within individual tumors). Tumor heterogeneity may be assessed in a single time point (spatial heterogeneity) or along the clinical evolution of GBM (longitudinal heterogeneity). Molecular methods may detect clonal and subclonal alterations to describe tumor evolution, even when samples from multiple areas are collected in the same time point (spatial-temporal heterogeneity). In GBM, although the inter-tumor mutational landscape is relatively homogeneous, intra-tumor heterogeneity is a striking feature of this tumor. In this review, we will address briefly the inter-tumor heterogeneity of the CNS tumors that yielded the current glioma classification. Next, we will take a deeper dive in the intra-tumor heterogeneity of GBMs, which directly affects prognosis and response to treatment. Our approach aims to follow technological developments, allowing for characterization of intra-tumor heterogeneity, beginning with differences on histomorphology of GBM and ending with molecular alterations observed at single-cell level.

## 1. Introduction

Gliomas are the most frequent primary malignant brain tumors, with glioblastoma (GBM) alone accounting for 15% of all central nervous system (CNS) tumors [1]. The optimum treatment with maximum safe surgical resection, followed by radiation with concurrent and adjuvant temozolomide (TMZ), significantly increases the overall survival (OS) of GBM patients compared to radiation monotherapy [2,3,4]. More recently, the addition of Tumor-Treating Fields (TTF) to adjuvant TMZ showed a significant improvement in both OS and progression-free survival (PFS) for patients with newly diagnosed GBM. After concomitant chemoradiation, the median OS was 20 months in the group of TTF plus TMZ and 16 months in the group with TMZ alone [5]. Nonetheless, the prognosis of GBM remains poor, with 5-year OS of only 7.2% [1]. One of the main reasons for this aggressive behavior is believed to be the intrinsic intra-tumor heterogeneity, conferring significant alterations in multiple hallmark cellular functions in cancer [6]. Differences in histomorphology, which for years justified the outdated eponym of “multiforme” to GBMs, are explained by a diversity of clonal and subclonal differentiated tumor cell populations, glioma stem cells (GSCs), and multiple non-tumor cells, such as endothelial and inflammatory cells, and other components of the tumor microenvironment (TME).

While heterogeneity is usually defined as “the quality or state of being diverse in character or content”, in clinical and translational research “Tumor Heterogeneity” usually encompasses both inter-tumor heterogeneity, i.e., population-level differences; and intra-tumor heterogeneity, which refers to differences within individual tumors. When evaluating individual patients, tumor heterogeneity may be assessed either in multiple samples collected at a single time point (spatial heterogeneity) or across samples collected over the clinical evolution, e.g., primary and recurrent tumors (longitudinal heterogeneity). The analyses of different clonal and subclonal tumor cell populations represent a historical record of alterations accumulated during a tumor history [7]. Therefore, studies of spatial heterogeneity may build the knowledge of the genesis and progression of GBMs, even when samples from multiple areas are collected in the same time point (spatial-temporal heterogeneity). On the other hand, studies of longitudinal heterogeneity allow for the detection of histopathologic and molecular changes at multiple time points to better understand tumor evolution and treatment resistance mechanisms. Exploring these two measures of tumor heterogeneity is the key to advancing personalized care for GBM patients.

Although intra-tumor heterogeneity is a striking feature of GBM, the inter-tumor mutational landscape of this tumor is relatively homogeneous compared with many other cancers [7,8,9], with GBM presenting a median of 2.2 somatic mutation per megabase 74 (Mb), compared to more than 8 in lung cancer and above 12 somatic mutations/Mb in 75 melanoma [9]. This allows for the classification of large groups based on common molecular alterations. In this review, we will briefly address the inter-tumor heterogeneity of CNS tumors leading to the current glioma classification. Next, we will take a deeper dive in the intra-tumor heterogeneity of GBMs, considering its consequences in prognosis, response to treatment, and importance in advancing personalized medicine. Our approach aims to follow the technological developments that allow for characterization of intra-tumor heterogeneity, beginning with differences on histopathological features of GBM until molecular alterations at single-cell level.

## 2. Materials and Methods

We performed searches in Pubmed and Google Scholar, using the keywords glioma OR glioblastoma AND heterogeneity until September of 2020. A large number of references identified in the search concerned themes beyond the scope of this review (for example, imaging studies), or simply showed results not related to tumor heterogeneity. Therefore, we manually selected 143 original articles and reviews written in English with an available abstract from 70 journals. No early date was excluded from the searches, in order to identify early studies that approached GBM heterogeneity with limited capacity, and whose findings were confirmed decades later by the novel technologies. All articles were included in a database containing information about the journals, type of heterogeneity, molecular methods used, main field of knowledge, and type of tumors included in the study. Although this is not intended to be a systematic review, we thought this analysis would help the readers have an overview of the evolution of the field in tumor heterogeneity in GBM.

Two authors (A.P.B. and B.E.S.) individually filtered abstracts deemed more pertinent to the theme of this review, and there was consensus on the inclusion of 70 articles. After careful reading of the articles, we excluded the ones that were not within the scope of the review. In course of preparing the current manuscript, we added references that were not identified or had not been published during the initial search process. Statistics and plot designs were performed with SPSS 25 (IBM) and R statistics [10].

**Figure 1:**
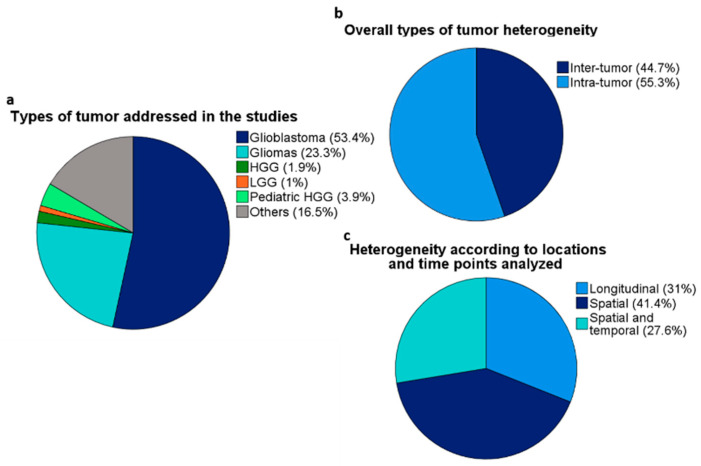
Overall profile of the selected articles cited in the review. (**a**) Type of tumors included in the studies. “Others” includes meningiomas, brain metastases, and other CNS tumors. (**b**) Types of tumor heterogeneity. (**c**) Types of tumor heterogeneity according to the origin of samples and time point of collection. Tumor evolution studies are counted in the “Spatial and temporal” category.

Ninety papers were deemed the most relevant for the theme, while the remaining references were indirectly associated with the theme of GBM tumor heterogeneity. The majority of the articles specifically addressed GBMs (51 out of 90 articles). However, articles including all diffuse gliomas (WHO grades 2–4), high-grade gliomas (HGGs—WHO grades 3 and 4), and lower grade gliomas (LGGs—WHO grades 2 and 3) were included because histopathological grade alone has decreasing importance as determinant for GBM diagnosis [11]. Some of the selected papers addressed pediatric HGGs, which are entities molecularly different from adult GBMs [11]; however, those references were included due to their importance in the field of neuro-oncology. Figure 1 depicts the overall profile of the papers.

**Figure 2:**
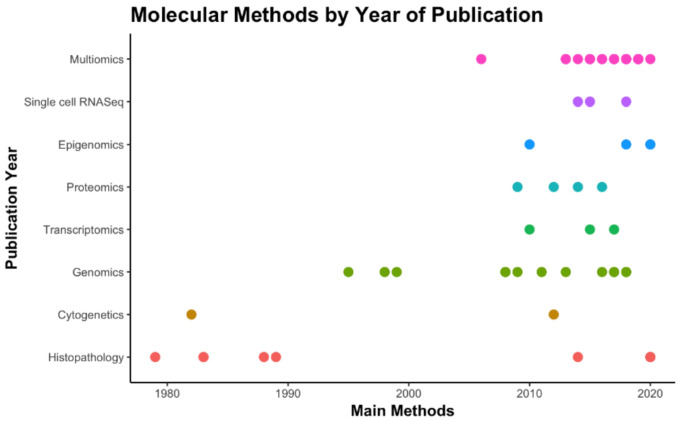
Timeline of methods by year of publication based on the articles selected in this review. “Histopathology” = H&E and IHC stains (the 1st WHO classification is from 1979). “Multi-omics” = studies with independent results from two or more high-throughput methods; studies with multiple methods for determination of groups in the study were not included in this count.

Remarkably, the exponential advances in molecular pathology over the last decade have resulted in multiple comprehensive papers, encompassing complex multi-platform analyses that are more likely to be accepted by premier journals (Figure 2). This may be explained both by the technological development and by the mounting interest in exploring tumor heterogeneity in GBMs to support advances in image exams and therapeutic approaches, discovery/validation of therapeutic targets and of prognostic/predictive biomarkers. Bioinformatics tools and algorithms have supported the analysis of the enormous amount of data produced by multiplatform high-throughput profiling studies, but are beyond our scope in this review.

## 3. Review

### 3.1. Inter-Tumor Heterogeneity: Classification and Grading of Gliomas

The glioma classification arose from neurosurgeons’ need to predict survival and propose therapeutic approaches for patients with brain tumors. In the 1920s, Bailey and Cushing classified brain tumors according to the embryological origin as astrocytomas (the most numerous group), ganglioneuroma, oligodendroglioma, ependymoma, pinealoma, and papilloma choroideum. GBM was identified as a less differentiated astrocytoma, then named “spongioblastoma multiforme” [12].

**Figure 3:**
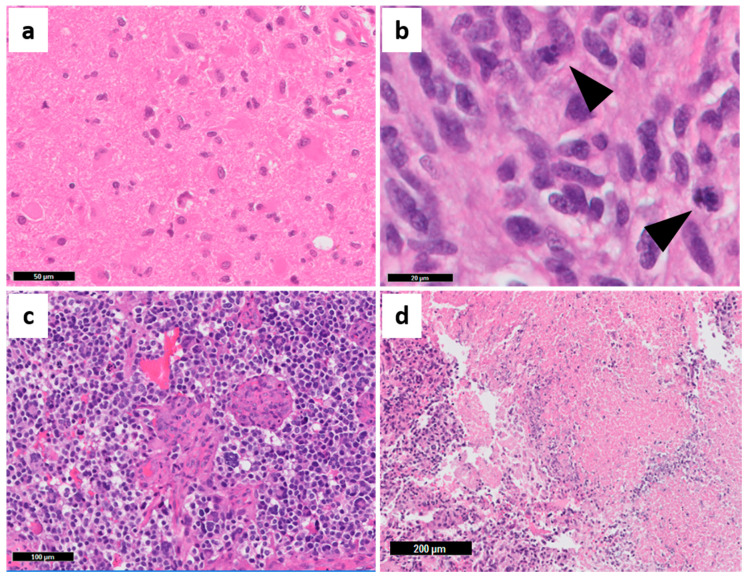
Saint Anne-Mayo histopathological criteria for astrocytoma grading. (**a**) Nuclear atypia in grade II—diffuse astrocytoma; (**b**) High mitotic activity (arrowheads) in grade III—anaplastic astrocytoma; (**c**) microvascular proliferation and (**d**) necrosis in GBM “multiforme”. Grade I gliomas are not part of this classification. Representative images captured from The Ohio State University (OSU) Tissue Archives.

Multiple histopathology-based grading systems sought to refine the prognosis of astrocytomas. The first World Health Organization (WHO) grading system in 1979 consisted of three histopathological grades, and did not include GBM as a diagnosis [13]. Later, the Saint Anne-Mayo system increased reproducibility of astrocytoma grading, by systematically evaluating four histopathological features: nuclear atypia, mitotic count, endothelial proliferation, and necrosis (Figure 3) [14].

The omics revolution of the past two decades shifted glioma classification from subjective histopathological criteria toward molecular profiling based on the genome, transcriptome, and methylome of individual tumors [15,16,17,18]. In this setting, as studies of brain tumors have always been at the forefront of biomedical research, GBM was one the first tumors to have its genome and transcriptome described by The Cancer Genome Atlas (TCGA) initiative [19,20]. Consequently, brain tumors were the first solid tumors to incorporate molecular features in their nomenclature. The current WHO 2016 classification still uses the histopathological grading system, but now molecular markers such as isocitrate dehydrogenase (*IDH*) 1/2 mutations and co-deletions in chromosomes 1p and 19q complete the integrated diagnosis of gliomas [21,22,23]. This resulted in three main glioma classes: IDH mutant, 1p19q codeleted (oligodendrogliomas), IDH mutant, 1p19q intact (astrocytomas), and IDH wild-type gliomas. Specific alterations are associated with each group, such as mutations on *CIC, FUBP1*, and *TERT* promoter; on *ATRX* and *TP53*; and on *EGFR*, *PDGFRA*, and *TERT* promoter, respectively [21,23]. In 2020, the consensus C-IMPACT-NOW 6 proposed an updated nomenclature for astrocytomas (oligodendrogliomas were not included) [11,24,25]. For *IDH* mutant astrocytomas, any combination of microvascular proliferation, necrosis (characteristics of anaplastic astrocytomas) or *CDKN2A/B* homozygous deletion now allows for the diagnosis of Astrocytoma, *IDH*-mutant, WHO grade 4—note the use of Arabic is now suggested, rather than Roman, numbers [11]. The diagnosis of GBM is now exclusive for *IDH* wild-type tumors with specific molecular features (gains in chromosome 7 and losses in chromosome 10, *EGFR* amplifications, and *TERT* promoter mutations), independent of the histopathological grade [11,26]. These changes are recommendations for the upcoming WHO classification predicted to be released in 2021.

Several other classifications based on the molecular profile of GBMs were proposed even before the WHO 2016 classification was released. Gene expression signatures categorize GBMs as proneural (enriched in *IDH* mutant tumors), neural, classical and mesenchymal, each with different prognosis, and histogenesis linked to, respectively, oligodendrocytes, neurons, astrocytes, and astroglial cells [18]; however, the possibility of the neural subtype corresponding to normal entrapped neurons was raised by subsequent authors [19,27]. DNA methylation pattern identified a group of GBMs and LGGs with hypermethylation at a large number of loci, called CpG Island Methylator Phenotype (G-CIMP), and favorable outcomes, which are virtually always related to the presence of *IDH* mutations [16]. Finally, a comprehensive analysis of these classifications defined three subgroups of *IDH* mutant gliomas (G-CIMP high—LGm1, G-CIMP low—LGm 2, and 1p19q codeleted—LGm 3) and four subgroups of *IDH* wild-type gliomas (classical-like—LGm4, mesenchymal-like—LGm5, and two rare subtypes—LGm6-GBM and PA-like), closely associated with transcriptional subtypes [15].

Not surprisingly, the molecular subtypes are closely associated with the main prognostic factors in GBM, *IDH* mutations and methylation of the promoter region of O^6^-methylguanine-DNA methyltransferase (*MGMT*) [21]. Virtually all proneural GBMs harbor *IDH* mutations and usually present G-CIMP, therefore reducing the prognostic/predictive power of *MGMT* promoter methylation in this group of tumors. Furthermore, *MGMT* promoter methylation is considered a predictive marker only in classical GBM, but not in the mesenchymal subtype [19].

**Figure 4:**
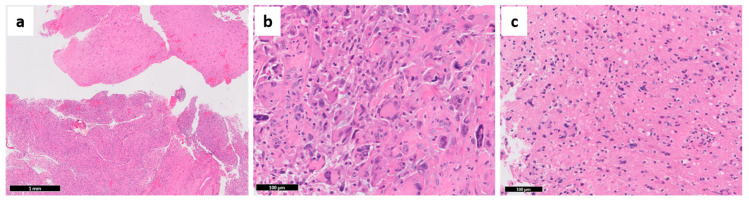
Additional features of glioblastoma. (**a**) Multiple histopathological grades can be seen in the same tumor—in this example, the upper fragments represent lower grade, while the bottom part depicts a fully developed GBM. (**b**) Extensive pleomorphism with giant cells may be seen, but the small cells are the most invasive component. (**c**) Inflammatory cells may invade GBM (same case as Figure 6a). Representative images captured from OSU Tissue Archives.

Because of the possibility of establishing GBM prognosis by other methods, despite its importance, the transcriptomic classification of GBMs has been used more in the research field than in the clinical setting. Recently, many authors have tried to develop and validate GBM sub-classifications using histopathological features and immunohistochemistry (IHC) approaches [27,28,29]. Initially, only moderate correlation of molecular features with the histopathological grades was observed [15]. However, in further analyses, small cell morphology and microvascular proliferation (Figure 3c) were associated with the classical subtype; oligodendroglial features with proneural subtype; pleomorphism and epithelioid cells, as well as inflammatory infiltration (Figure 4b,c and Figure 6a–c), with the mesenchymal subtype [27]. Moreover, a strong correlation between the GBM transcriptional subtypes and IHC profile was demonstrated, with higher expression of ASCL1, OLIG2, and PDGFRα in proneural GBMs; EGFR in classical GBM; and p53, pNDRG1, YKL40, and MET in mesenchymal GBM, which were able to predict the molecular subtypes with high efficacy using machine learning tools [27]. Other authors characterized a classical/proliferating profile (overexpression of EGFR and Olig2, and high proliferative activity), and a mesenchymal/microglial profile (overexpression of ALDH1A3 in tumor cells and Iba-1 in microglia) by IHC, which were closely related to the transcriptional profiles with similar names [29]. A subset of GBMs contained areas of both profiles (“subtype heterogeneous”) and had worse OS. Their results also confirmed the inflammatory profile of the mesenchymal subtype, which predominates in recurrent GBMs [29]. With the growing need for molecular tests to establish the final diagnosis of CNS tumors [11,21], the use of an IHC panel makes the subclassification of GBMs more speedy and affordable for oncology services worldwide.

In summary, glioma grading and classification is a complex, evolving field. Inter-tumor heterogeneity studies are the basis of the WHO classification and its regular updates. These studies need a large number of samples, but allow for the observation of common histopathological and/or molecular characteristics, as well as the discovery and validation of prognostic/predictive biomarkers. Nevertheless, noticeable variability in outcomes is still seen among individuals of a single group of tumors. Therefore, closer analysis of individual tumors is necessary to assess how intra-tumor heterogeneity influences the outcomes and responses to treatment of GBM patients. In the next sections, we will review how different methods have helped advance the understanding of tumor biology and how intra-tumor heterogeneity studies are paving the way to a more individualized care for patients with gliomas.

### 3.2. Intra-Tumor Heterogeneity

#### 3.2.1. Spatial Heterogeneity and Spatial-Temporal Histomorphology-Based Methods

Although histopathology was the foundation for gliomas grading and classification, hematoxylin and eosin (H&E) analyses are intrinsically subjective and highly dependent on the representation of the tumor. As an example, several features used for astrocytoma grading are prone to subjectivity, resulting in up to 20% inter-observer disagreement [30]. One of the reasons for this discrepancy is that 62% of all gliomas retain areas histologically representative of grades 2, 3, and 4 within a single tumor, which enhances the chance of grade underestimation in small tissue fragments (e.g., stereotactic biopsies) (Figure 4a) [30,31]. Despite that, histopathology has guided GBM research, and several methods of molecular evaluation, such as fluorescence in situ hybridization (FISH) and IHC, require correlation with histopathological features. These methods have greatly benefitted from the development of tissue microarrays (TMAs) [32]. TMAs enable the simultaneous study of dozens of formalin-fixed, paraffin embedded (FFPE) specimens in a single block, supporting back-to-back comparisons of multiple tumors, multiple areas from a single tumor, and of matched pairs of primary and recurrence tumors.

Despite the limitations, several histopathological features were associated with response to different treatments. Tumors with high cellularity were negatively associated with survival in patients with no adjuvant treatment. Not surprisingly, high cellularity presented a positive correlation in patients treated with radiation plus 1,3 bis (2-chloroethyl)-1-nitrosourea (BCNU), reflecting the susceptibility of tumor cells to chemo-radiation. Necrosis, microvascular proliferation, and infiltration of inflammatory cells were not associated with survival or response to treatment [33]. Correlative studies with autopsy material and computerized tomography (CT) images, indicated that small anaplastic and small fibrillary cell populations (not the pleomorphic cells—Figure 4b) enhanced GBM invasiveness and mass effect, but also improved response to treatment. The authors also described different cell densities in the necrotic center, the contrast-enhancing rim, and in the perilesional area [34,35], which are radiological regions of interest in all image exams in the context of neuro-oncology. Although the radiological aspects of glioma heterogeneity are beyond the scope of this review, it is important to note that advances in neuroimaging techniques such as magnetic resonance image (MRI) sequences improved the guidance for stereotactic biopsies, and predictions based on texture analysis and machine learning algorithms are advancing radiomics to a new field of radiogenomics [36,37,38].

**Figure 5:**
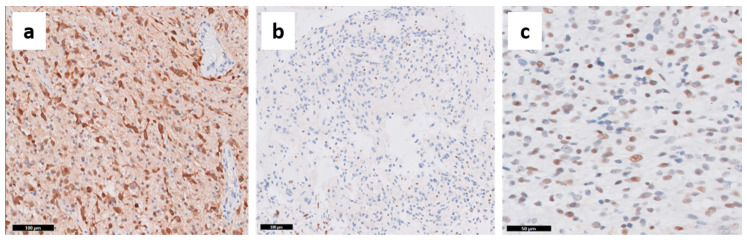
Molecular features of GBM assessed by immunohistochemistry. (**a**) IDH1 R132H, (**b**) ATRX loss (positive stain is seen in endothelial cells), and (**c**) p53 expression are diffuse in the tumor. Representative images captured from OSU Tissue Archives.

Unfortunately, some high-throughput molecular techniques demand tissue dissociation, which results in the loss of important information on the cellular and subcellular expression of altered genes. Morphology-based studies with IHC may detect the consequences of multiple molecular alterations in tumor cells. For example, gene mutations with gain of function and amplifications result in increased protein expression, while reduced expression may follow loss-of-function mutations and DNA methylation. Several authors have extensively demonstrated the expression of GBM markers, such as EGFR, MGMT, IDH1 R132H, and ATRX, in tumor cells using IHC, which is much more applicable for neuropathology routine [39,40,41,42,43,44,45,46]. With systematic evaluation of IHC stains, several authors showed that while endothelial cells do not express EGFR, regional differences in EGFR staining in GBM tumor cells may be explained by different levels of *EGFR* amplification [45,46], with higher EGFR amplification being associated with high proliferative activity, higher mutational burden, and shorter OS [45]. Heterogeneous MGMT expression in gliomas including positive stain in endothelial cells and lymphocytes has been described for decades [40,42,44]. Although MGMT IHC is not the gold standard prognostic test for GBMs, it is suggested that MGMT expression assessed by IHC can refine the predictive power of DNA methylation tests used in the clinical setting [41,43]. Our group assessed MGMT and GFAP co-expression with fluorescence IHC (Automated QUantitative Analysis—AQUA) and demonstrated that IHC may be a better prognostic test than the DNA methylation tests for patient stratification in GBM clinical trials [39]. On the other hand, because *TP53*, *ATRX* and *IDH* mutations are early events in gliomas [23], protein expression assessed by IHC is either diffusely lost (ATRX) or diffusely present (protein IDH1 R132H, p53) in tumor cells (Figure 5). ATRX has shown heterogeneous expression in only about 20% of GBMs, associated with higher frequency of *EGFR* amplifications [47]. Whereas p53 IHC staining is not sensitive nor specific for TP53 mutations [21], the diffuse staining reflects its early occurrence in gliomagenesis.

##### Chromosomal and DNA Ploidy Analyses

Early studies associated differences in karyotypes of tumor cell clones with specific morphology and growing rate. Fast-growing fibroblast-like cells showed hyperploid karyotypes, while slow-growing astrocyte-like cells presented near-diploid karyotypes [48]. More recently, the combination of DNA ploidy and stem cell markers at single-cell level provided new insights on the evaluation of tumor clones. Considering genomic instability is a hallmark of GBM [49], DNA ploidy identified a subset of GBMs that displayed both pseudodiploid and aneuploid tumor cell clones, characterizing the clonal evolution process towards aneuploidy. Although both pseudodiploid and aneuploidy clones were able to form spheroids in vitro, the aneuploid clones instigated more aggressive tumors in vivo, with shorter OS [50]. To investigate stemness of these tumor cell clones, the authors evaluated the expression of several cancer stem-cells (CSCs) cell-membrane markers. Both diploid and aneuploid clones expressed CD56, CD90, and CD29 in similar levels; A2B5 expression was stronger in the diploid compared with the aneuploid fraction. However, CD133 and CD15 were heterogeneous, enriched either in the aneuploid or in diploid fraction tumor clones in different tumors. These findings suggested that in polygenomic GBMs the tumorigenic potential depends on DNA ploidy of CSCs [50].

At chromosomal level, early cytogenetic analyses of multiple samples obtained from one tumor diagnosed as “low grade oligoastrocytoma” demonstrated a ubiquitous tumor cell clone with gains in chromosome 7 (47XY, +7), while cells with concurrent chromosome 7 gains and chromosome 9 losses (47,XY,+7/47,idem,+del(9)(q21.2)/47,idem -9,+ 16) were deemed a subclonal population [51]. Intriguingly, the region with both chromosomal alterations exhibited higher pleomorphism and mitotic activity [51]. C-IMPACT-NOW-6 recently incorporated these early chromosomal alterations to the updated glioma classification independent of histopathology [11]. Therefore, although the chromosomal changes were focal, that tumor could be now classified as grade 4 astrocytoma or GBM (depending on *IDH* status), even in the absence of microvascular proliferation or necrosis [11,24,51]. Several further studies with comparative genomic hybridization (CGH) and laser cytometry showed that DNA aneuploidy, chromosome 7 gains, and losses in chromosomes 9, 10, 13, and 22 were diffuse in grade 3 and 4 gliomas, suggesting that genetic instability and cytogenetic heterogeneity, constitute early alterations in glioma progression [26,52,53]. Yet those findings were not associated with any histopathologic features [52]. Remarkably, cytogenetic evaluation of TME components, specifically endothelial cells showed that intra-tumor endothelial proliferation displayed unique chromosomal changes, with modest overlapping with the alterations observed in the tumor cells. This is believed to be due to tumor endothelial cells being generated by transdifferentiation of glioblastoma cells [54]. Only one of the tumors in the cohort exhibited the characteristic chromosome 7 gains observed in GBMs [55]. In single-cell studies, chromosome 7 gains and chromosome 10 losses were common to all 430 analyzed cells from a small GBM series. Other chromosomal alterations were observed in the cohort; however, this method may underestimate the presence of focal alterations occurring in very small percentage of tumor cells [56].

Given that alterations in receptor tyrosine kinase (RTKs) are a hallmark of 50% of GBMs [19,20], multiple groups have investigated the frequency and distribution of RTK amplifications with array comparative genomic hybridization (aCGH) with single-nucleotide polymorphism (SNP) analysis of specific gene loci in chromosomal regions [56,57,58]. *EGFR* amplifications were more frequent in GBMs than in LGGs, while *PDGFRA* amplifications were more homogenous among different tumor grades [57]. In addition to previously reported intra-tumor heterogeneity of *EGFR* amplifications [45,46], a subset of GBMs displayed mosaic amplifications in multiple RTKs, mainly involving *EGFR, PDGFRA*, and *MET*, with *EGFR* and *PDGFRA* co-amplification being the most common combinations, followed by *EGFR* and *MET* co-amplification. Rather than co-occurring in single cells, those RTKs were amplified in different cell populations, which overexpressed exclusively the correspondent protein [57,58]. Importantly, *EGFR* amplifications were associated with aggressive phenotype, represented by poor vascularization and small cell morphology, the more invasive cell type as previously described [34,35]. Conversely, *PDGFRA*-amplified cells were significantly associated with fibrillary morphology and more vascularized areas [57]. Post-mortem analysis of one GBM with a mosaic pattern of RTK amplifications showed that tumor cells with *PDGFRA* amplifications were limited to the bulk of the tumor, while *EGFR* amplifications were observed in the cell population present at areas of infiltration [58]. Single-cell RNA sequencing has also confirmed the possibility of mosaic expression of multiple RTK amplifications in *EGFR, PDGFRA, PDGFA, FGFR1, FGF1, NOTCH2,* and *JAG1* in GBMs [56].

More recently, gene amplifications were linked to extrachromosomal DNA (ecDNA) elements, which likely give origin to different tumor cells sub-clones. The presence of ecDNA was more frequent in *IDH*-wild-type, compared to *IDH*-mutant GBMs, and targeted several oncogenes, namely *MET, EGFR, CDK4, PDGFRA*, and *MYC*, were maintained in recurrent tumors after adjuvant treatment [59]. The therapeutic implication of ecDNA elements as a mechanism of gene amplification was demonstrated in vivo. Tumors with *MET* amplification associated with high-frequency ecDNA elements had worse response to treatment with a MET inhibitor than the tumors with *MET* amplification without ecDNA elements, even though both presented MET overexpression, thus suggesting that ecDNA is a significant mechanism of treatment resistance to be considered in further studies [59].

##### Genomic and Transcriptomic Analyses

As the molecular classification of GBMs relies on both genomic and transcriptomic data, virtually all current GBM studies perform DNA sequencing and/or gene expression data for cohort characterization. Therefore, whole genome sequencing (WGS), whole exome sequencing (WES), RNA sequencing, gene expression arrays, and other methods are ubiquitous in glioma research [23,59,60,61,62,63,64,65]. Importantly, the development of cell sorting through bar coding techniques enabled the evaluation of gene expression at single cell level [56,62,66,67].

In an evolutionary tree, truncal mutations are observed in every sequenced cancer cell, while branches represent subclonal mutations, present in only a subset of cancer cells. Although those are concepts closely associated with the definition of clonal and subclonal mutations, respectively, the distinction between truly clonal from pseudoclonal mutations is dependent on the number of tumor regions sequenced [7]. Suzuki et al. described regional and temporal heterogeneity based in the genomic profile of multiple LGGs areas, showing that approximately 60% of mutations were branch (subclonal) mutations, while only 10% were defined as truncal (clonal) mutations [23]. Based on these findings, this group was one of the first to describe the putative order of molecular alterations in gliomas incorporated in the WHO 2016 classification with *IDH* point mutations, *TERT* promoter mutations, and 1p19q co-deletion as early events. These events are followed by frequent mutations in *ATRX/TP53* (in 1p19q intact tumors) or *FUBP1/CIC* (in 1p19q co-deleted tumors), which affect different tumor cell sub-clones, reflecting on the concept that clonal evolution of tumors occurs in both space and time [23].

Even before the genomic landscape of LGGs was described, GBM spatial-longitudinal evolution was inferred based on the frequency of the alterations between the samples by assessing DNA somatic copy number alterations (CNAs), single-nucleotide variants (SNVs), and gene expression profiles in multiple tumor areas [68]. This was one of the first articles to observe individual clones with different transcriptional subtypes coexisting within the tumors. In addition, early CNAs on chromosomes 7, 9, and 10, loci of several GBM drivers (*EGFR, CDK6, CDKN2A/B, MET*, and *PTEN*) were diffusely seen in the tumors. Subsequent alterations affected genomic regions containing *PDGFRA* (chromosome 4), *PTEN* (chromosome 10), and *TP53* (chromosome 17), and were observed in limited/unique areas. With that, the authors achieved temporal and spatial reconstruction of GBM ontogeny [68]. In fact, the genetic distance between tumor areas was observed by other authors, and is thought to be correlated with the physical (Euclidian) distance between the tumor areas [64].

In pediatric tumors, Hoffman et al. described spatial heterogeneity of midline HGGs (mHGG), which included tumors with and without K27M mutations in *H3F3A* or *HIST1H3B* (collectively called H3K27M). In diffuse intrinsic pontine glioma (DIPG—also called diffuse midline gliomas), H3K27M mutations, as well as *ACVR1, FGFR1, MET,* and *PIK3CA* mutations, were clonal. Subclonal gene mutations and amplifications involved *PDGFRA, ATRX, BCOR*, and *MYC* [60]. On the other hand, one pediatric case of mHGG H3- wild-type showed diffuse (probably clonal) *ATRX* and *TP53* mutations and *PDGFRA* amplification [60]. Although these changes are frequent in adult gliomas following an initiating *IDH* mutation [23], in pediatric patients they are probably initiating events, since *IDH* mutations are rare in DIPG and pediatric HGG [69].

Vinci et al. further confirmed the clonal nature of H3K27M, *ATRX*, and *NF1* mutations, as well as the subclonal expansion of cells bearing mutations in *TP53, BRAF, PDGFRA*, among others, in pediatric GBM (pGBM) and DIPG [61]. The authors identified differences in cell morphology, growth, migration, and invasion directly associated with genomic profile of the tumor sub-clones, even when arising in rare sub-clone variants. For example, rare mutations in the histone H4 methyltransferase *KMT5B*, enhanced invasiveness, and sensitivity of tumor cells to PARP inhibitors in vitro and in vivo. Co-culture of *KMT5B* mutant and *KMT5B* wild-type cells and monoculture using conditioned medium from the parental cells (bulk cells) showed significant enhancement of migration and invasion compared with single clone culture, both in vitro and in vivo, and suggested the importance of cell-cell interaction and paracrine signaling as mechanisms in tumor development. This suggests that in DIPG, multiple sub-clones cooperate to enhance tumorigenic phenotypes [61]. The concept of ‘cooperative invasion’ had previously been described in other cancers and in GBM [70].

In adult GBM, multiple studies have demonstrated heterogeneous transcriptome profiles within single GBMs using single-cell RNA sequencing [56,62,71]. Patel et al. defined four meta-signatures in tumor cells enriched for genes associated with cell cycle, hypoxia, and complement/immune response that correlated with the molecular subtype of GBM in single cells, which did not always correspond to that assigned in the bulk tumor [56,62]. The gene meta-signature associated with cell cycle was expressed in a small proportion of cells in each tumor and was negatively correlated with stemness and with hypoxia gene signatures. This stemness meta-signature was observed in cells of proneural and classical subtypes, but not in the mesenchymal subtype. The variability of the cell cycle meta-signature among tumor cells may be associated with variations in the TME [56]. Finally, multiple groups have reported the occurrence of hybrid tumor cells expressing both classical and mesenchymal or proneural and classical signatures that are associated with worse OS [56,71]. Intra-tumor heterogeneity defined in single-cell analyses may present prognostic implications. Upon the evaluation of bulk proneural GBMs, the authors observed the proportion of tumor cells of alternate subtypes influences the clinical outcome, with increased heterogeneity being associated with worse OS [56]. Meyer at al. demonstrated the presence of TMZ-resistant clones in a GBM not submitted to previous treatment, suggesting that resistant clones do not arise solely from therapeutic selection. Similarly, a TMZ-sensitive subclone was isolated from an otherwise recurrent GBM, which presented several TMZ-resistant clones with significant difference in the sensitivity to multiple target therapies [71]. Recently, Akgul et al. also reported that multiple molecular differences in subclones reflected different responses both to the standard GBM treatment (TMZ + radiation) and to multiple targeted therapies [62]. These findings justify the concurrent use of different combination therapies, especially in recurrent GBMs [62,71].

Because advanced technologies may detect subtle differences between tumor cells, using multiple samples from single tumors can allow for the study of spatio-longitudinal heterogeneity [23,26,50,53,67,72,73]. In this setting, studies on multicentric/multifocal GBM (M-GBM) and M-LGG have also shed light on the clonal evolution of these tumors [74,75,76]. M-GBMs and single GBMs share common molecular alterations. Similar to other reports, in M-GBMs, chromosomal alterations are early events, as shown by shared CNAs present in all areas of the tumors. On the other hand, specific gene mutations unique to different foci point toward a parallel genetic evolution from a common tumor precursor clone (early separation of cell clones) [74]. Hayes et al. described different *IDH1* point mutations (R132H and R132C) in different tumor areas of one M-LGG. The patient had a germline mutation on *TP53* present in only in the tumor foci with the non-canonical *IDH1* mutation. In another patient, two foci of M-LGG presented both grade 2 and 3 histopathologic grades. In this patient, multiple *TP53* and *ATRX* point mutations were conserved among samples, but distinct between the two grades. Finally, two patients in this series presented M-LGG with divergent ATRX/1p19q status between the tumor foci, challenging the discouragement of the diagnosis of “oligoastrocytoma” in tumors with mixed histopathological aspects [75]. These findings confirm the divergent evolution in multifocal tumors and the need for careful evaluation of small samples that may misrepresent the whole tumor.

##### DNA Methylation Analyses

Epigenetic characterization of GBMs has provided great insight into inter-tumoral heterogeneity and glioma classification and prognosis [15,16]. DNA methylation of CpG islands in the gene promoter are associated with repression of gene expression [77]. These bear a close relationship with hypermethylated status conferred by *IDH* mutations in gliomas [15,16]. The methylome of tumors has been integrated in most heterogeneity papers using high-throughput methods, especially for temporal heterogeneity studies. DNA methylation has also advanced the knowledge in intra-tumor spatial heterogeneity, especially in the characterization of CSCs and inflammatory cells [78,79,80,81]. Notably, differences in DNA methylation between tumor areas as close as 5 mm apart were observed, both in *IDH* mutant and *IDH* wild-type gliomas [64]. For specific information about DNA methylation and its relationship with other epigenetic modifications in cancer, we recommend a recent review covering this topic [77].

Comparisons between the DNA methylome of secondary astrocytomas *IDH* mutant grade 4 (former *IDH* mutant GBMs), and their matched primary LGG found that the G-CIMP profile present in all primary tumors was maintained in the recurrences. However, the malignant progression of gliomas toward GBM showed loss of DNA methylation specifically in cell cycle-related genes [73]. The changes in the DNA methylation profile reflected the phylogenetic tree of gene mutations among tumor areas from a single tumor and between matched primary and recurrent tumors. However, in one patient with three LGG recurrences, the genomic changes occurred prior to (but in co-dependency with) epigenomic alteration in the glioma recurrences with grade progression. Therefore, the phyloepigenetic tree was slightly divergent from the phylogenetic tree of the same samples [73].

Klughammer et al. used reduced representation bisulfite sequencing (RRBS), a novel method that “allows for individual cell assessments, without the need for single-cell sequencing, and precludes the need for RNA extraction”, to assess temporal and spatial heterogeneity in newly diagnosed and recurrent GBMs [72]. The authors reproduced the transcriptome subclassification of GBMs with high confidence, including the identification of multiple subtypes in tumor cell clones [56,71], and showed significantly hypermethylation of chromatin binding proteins (*CTCF, EZH2*, and *KDM4A)* and reduced methylation of stemness regulators (*OCT4, NANOG, SOX2*) in the mesenchymal subtype. Their pathway analysis identified enrichment of neural development and apoptosis signaling among genes whose promoters gained DNA methylation during disease progression, while genes whose promoters lost DNA methylation were enriched in the Wnt signaling pathway and T-cell activation [72]. In addition, using machine learning methods, specific DNA profiles were associated with GBM subtypes, the presence and density of inflammatory cells (macrophages and lymphocytes), histopathological features (necrotic area, cell morphology, and mitotic activity), and prognosis. For example, mesenchymal GBM was more associated with infiltration of inflammatory cells, lower cell density and extensive necrotic areas. These characteristics were also more significant in recurrent tumors, compared to primary tumors [72].

Pangeni et al. characterized the DNA methylation profile of GSCs, and observed higher frequencies of methylated CpGs than GBM bulk tumor cells, especially in mesenchymal GBMs. Specific DNA methylation signatures were observed in CSCs and in GBM bulk tumor, with only few overlappings, in both proneural and mesenchymal GBMs [82]. Peculiarly, these authors described predominance of global hypomethylation events in proneural GBMs, which are usually *IDH*-mutant, therefore hypermethylated tumors. However, the proneural GBMs of their series were reportedly *IDH* wild-type tumors, less likely to present high G-CIMP [16,82]. More specifically, because GSCs are pluripotent cells with self-renewal and proliferation properties, the expression of *EZH2,* a protein related to maintenance of stemness is of high importance, especially in H3K27- mutant tumors. Overexpression of *EZH2* in HGGs is associated with worse OS. This gene is part of the polycomb repressive complexes involved in the epigenetic modulation of GSCs, the most studied epigenetic modulators in GSCs, and is therefore a potential therapeutic target [80].

Recently, Feng et al. proposed a signature based on the methylome profile composed by five candidate probes closely associated with the DNA damage repair function as an independent predictive marker for radiation therapy in gliomas [79]. Similarly, Wang et al. proposed a methylation-based classification of GBMs to predict response to immunotherapy [81]. They correlated DNA methylation and gene expression to define two clusters of GBMs with different prognosis, expression of immune checkpoint molecules, and response to immunotherapy, independent of other clinic prognostic factors. Six novel candidate genes (*ANKRD10, BMP2, LOXL1, RPL39L, TMEM52*, and *VILL*) were selected-based in survival analyses, to propose a reliable prognostic model, which resulted in an effective nomogram for survival prediction in GBM that was likely the first to incorporate global methylation signature [81].

##### Tumor Microenvironment: One of the Pillars of Intra-Tumor Heterogeneity

**Figure 6:**
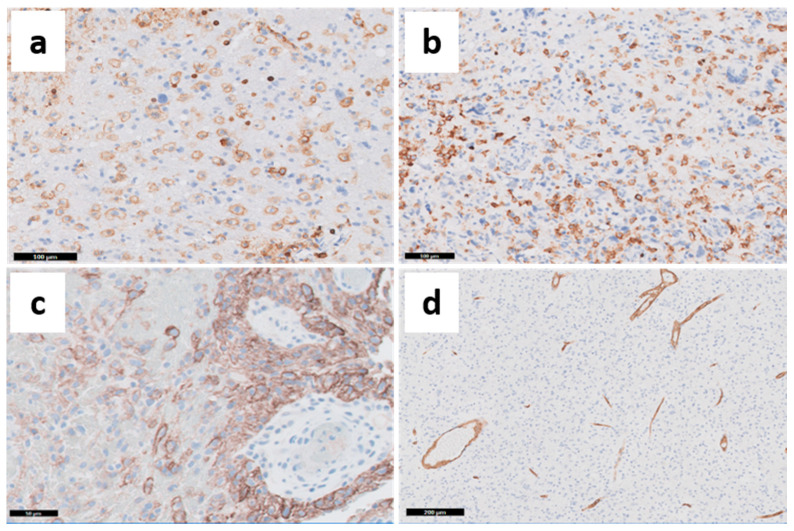
Characterization of TME with IHC markers. (**a**) CD45 expression: hematopoietic cells (both lymphocytes and macrophages). (**b**) CD163 expression: M2 macrophages. (**c**) PDL-1 expression: presence of positive tumor cells in the perivascular space. (**d**) CD34 expression: endothelial cells. Representative images captured from OSU Tissue Archives.

Besides multiple tumor cell clones, other cell populations including normal glial cells (astrocytes and oligodendrocytes), pericytes, and mainly endothelial and inflammatory cells (microglia/macrophages and lymphocytes) are an essential part of the tumor heterogeneity in GBMs (Figure 6) [70].

The interactions between the TME components with the tumor cells are increasingly being explored and may determine response to immunotherapy and enable the development of new targeted therapies. Histologically, it is not difficult for the pathologist to identify and characterize, with H&E and IHC stains, the presence of microvascular proliferation and inflammatory infiltrate in GBMs. The characterization of macrophages relies in the expression of CD68, CD163 (marker of M2 polarized macrophages—Figure 6b), and IBA-1, among others. The difference between macrophage and resident microglia is based on high and low expression of CD45 (Figure 6a), respectively, and CD11b expression by microglia. The lymphocyte population is characterized by the expression of CD45, CD3, CD4, CD8, FOXP3, and more recently PD-1 [83,84]. The analysis of inflammatory cells has gained momentum with the development of cell sorting methods and single-cell analyses, improving the potential for use of immunotherapy and identification of new therapeutic targets. In addition, the analysis of cytokines and their effects in tumor initiation and progression has advanced with other methods not explored here, but is reviewed by other authors [85].

Several authors have identified that GBMs are 30–50% composed of microglia/macrophages (Figure 6b) [84,86,87,88]. However, tumor-associated macrophages (TAMs) do not retain the ability to activate immune response through T-cells, because while expressing MHC class II, TAMs lack the expression of co-stimulatory factors (CD80, CD86, CD40) and most cytokines (mainly IL-1, IL-10, and TNF-α) needed for adaptive immunity [84]. Over the years, the more comprehensive understanding of macrophage functions led to subclassification according to their pro-inflammatory and anti-tumor properties as M1 and M2, respectively, observed in vitro [89,90]. However, these definitions do not translate well to the in vivo setting, and TAMs express marker characteristics of both M1 and M2 phenotypes, with the ability to transition between phenotypes, depending on the interactions with the TME [83]. Increasing evidence suggests that TAMs promote tumor invasion and growth (through several factors released from microglia, such as STI-1, EGF, CSF-1, CCL2, and TGF-β), and angiogenesis, through VEGF expression. The expression of TGF-β by TAMs is also associated with increased GSC invasiveness, whereas GSC recruit TAMs more efficiently than differentiated tumor cells. An in-depth review of the complexity of the interactions between the different TAMs phenotypes and between TAM and other components of gliomas may be seen in other reviews [83,89].

Tumor-infiltrating lymphocytes (TIL) usually correspond to up to 10% of glioma content, represented by T-cells, natural killer (NK) cells and B lymphocytes [83,87,88]. Nevertheless, their presence is so meaningful that it is the basis for another classification of solid tumors as “hot” (inflamed) and “cold” (non-inflamed) tumors based on an immunoscore initially developed for colorectal cancer that accounts for the presence of CD3 and CD8, markers of T-lymphocytes, in the tumor core and margins [91,92]. In general, GBM is considered a “cold” tumor, with important diagnostic/prognostic and therapeutic consequences [93]. Converting GBM from a “cold” to a “hot” phenotype is one of the goals in improving response to immunotherapies. For example, the metabolic product of mutant *IDH1/2*, 2-hydroxiglutarate (2-HG), suppresses antitumor activity of tumor-infiltrated cytotoxic T-cells in vivo [94], and therefore, *IDH* mutations are, per se, targets for new vaccines. In addition, activation of the PI3K pathway in GBM cells results in the expression of the immune checkpoint ligand PD-L1 (Figure 6c); additionally, activation of the Ras–MAPK pathway induces the expression of IL6 and TGF-β, which enhances the recruitment of TAMs and inhibits both dendritic cells (DC) and lymphocytes infiltration into the tumor. This event is now seen as a potential avenue for development of multiple targeted interventions [93]. Single-agent neo-adjuvant immunotherapy with check point inhibitors (PD1 axis inhibitor, nivolumab) has failed to show clinical benefits in GBM [95]. However, neoadjuvant use of PD-L1 inhibitor resulted in significant modification in the TME towards a stronger anti-tumor immune response [95,96].

TILs may affect the outcomes of GBMs subtypes [67,72,78,82,97,98]. Wang et al. described the specific associations of transcriptomic subtypes of GBM with immune cells gene signatures [67]. Proneural GBMs were associated with a reduced CD4+ T-cells gene signatures, the classical GBMs were associated with an enhanced dendritic cell (DC) signature, and the mesenchymal GBMs were associated with enhanced M1 macrophage and neutrophil, but decreased NK cells gene signatures [15,67]. Other authors have reported increased infiltration of immune cells in mesenchymal type GBM compared to proneural and classical subtypes, using different assessment methods [72,78]. More specifically, mesenchymal tumors showed the highest amount of tumor-infiltrating CD3+ and CD8+ T-cells, while *IDH* mutant proneural tumors showed the lowest amount of CD3+ and CD8+ T-cell infiltration. Expression of CD68, FOXP3, and PD-1 did not show differences between the subtypes [78]. Importantly, these authors compared matched samples of patients before and after treatment with TMZ + radiation and an experimental DC vaccination. While CD3+ T-cells did not show differences over time, there was a reduction in CD4+ T-cells and an increase in CD8+ PD-1+ T-cells in all methylation types. Macrophage infiltration did not show differences between the subtypes with a general IHC marker (CD68) unable to show differences in M1/M2 macrophages. Although direct effects in OS were not detected, the findings confirm that the more aggressive mesenchymal GBMs presented higher T-cell infiltration, conferring a more immunogenic nature to those tumors [78]. To demonstrate the interaction between glioma tumor cells and immune cells, Zhai et al. showed that mRNA expression of Indoleamine 2,3-dioxygense 1 (IDO1), an immunossupressive enzyme, increases in tumor cells with histologic grade in gliomas. IDO1 showed increased expression in mesenchymal and classical GBMs compared with proneural GBMs, and was higher in *IDH* wild-type than in *IDH* mutant gliomas. Thus, high IDO1 expression was related to worse OS. Importantly, IDO1 expression in tumor cells increased with the increased expression of markers of TAMs (such as HLA-DRA and CD163) and neutrophils (for example, CD11b and CD16), and with multiple lymphocitic markers, including FOXP3, PD-1 and PD-L1, suggesting that T-cells directly regulate IDO1 expression in human GBM. Unfortunately, the IHC detection of IDO1 was minimal, and the evaluation of this important marker was only possible with mRNA measurements [98]. 

In pediatric HGGs, Bockmayr et al. identified four groups of tumors on the basis of the gene signatures of infiltrating immune cells—vascular (immune 1); monocytic and stromal (immune 2); monocytic and T-cell dominant (immune 3); and antigen-presenting cells (APC), NK cells, and T-cells (immune 4)—that were associated with the transcriptomic molecular subtypes. The vascular signature was associated with the classical subtype, while the mesenchymal subtype was associated with monocytes and T-cell gene signatures (immune 2 and 3). Interestingly, the immune 4 signature (innate immunity) was enriched in Pediatric HGGs with G34 mutations, a histone H3-mutated tumor with better prognosis than other pediatric HGGs [97]. This reflects the anti-tumor effects of the innate immune response.

Tumor immunology is an extremely complex field, with the same immune cells being able to perform anti- and/or pro-tumoral functions. Therefore, the development of combinatory therapies with novel drugs targeting multiple candidate molecules along the chain of events in the inflammatory response to tumors, rather than use of single-drug approaches, is an urgent need in GBMs.

A summary of the histopathological, immunohistochemical, and molecular features of grade 4 *IDH* mutant astrocytomas and different subtypes of GBMs is shown in Table 1.

#### 3.2.2. Longitudinal Heterogeneity

Although maximum surgical resection has provided significant survival benefits for patients with recurrent GBM [99,100], some controversy over operating on patients with recurrent tumor still exists. With this in mind, studies on GBM longitudinal heterogeneity are needed to better understand the differences between primary and recurrent GBMs. Two recent comprehensive articles have begun to look at this by comparing patient’s samples collected at multiple time points and have produced valuable findings regarding GBM evolution [53,101].

The Glioma Longitudinal Analysis (GLASS) consortium characterized the largest cohort of matched primary and recurrent gliomas, including all three WHO classes [101]. Overall, the mutational burden was low in primary gliomas of all classes, but increased in 70% of recurrent tumors. Hypermutational state was associated with treatment with alkylating agent, and significantly more frequent in *IDH* mutant-noncodel gliomas (47%), than in *IDH* mutant-codels (25%) and *IDH* wild-type gliomas (16%). The clonal structure at the primary tumors mostly persisted into recurrences, and the neutral-to-neutral trajectory (random mutations that are not subject to selection) was the most common evolutionary trajectory overall. Nevertheless, selection model was more frequent in *IDH* wild-type gliomas at any time point. Selection evolution at recurrence was associated with shorter OS than neutral evolution in all groups [101]. The order of *IDH1*, *ATRX*, and *TP53* mutations followed previous reports [23]. On the other hand, in *IDH* wild-type tumors, their findings indicated chromosome 10 deletions as earlier events than chromosome 7 amplifications. A gross comparison of all shared mutations revealed that cancer cell fractions (CCF) increased in recurrent *IDH*-mutant-noncodel gliomas and decreased in recurrent *IDH* wild-type glioma, which suggests, respectively, reductions and increases in intra-tumor heterogeneity in recurrent tumors [101]. Concerning specific drivers of glioma progression, recurrent *IDH* mutant-noncodel gliomas presented an enrichment in *CDKN2A* homozygous deletions, with subsequent DNA aneuploidy and genetic instability that were associated with shorter OS [101].

Finally, because 80% of LGGs harbor *IDH1* or *IDH2* mono-allelic mutations [98], secondary GBMs (GBMs arising from previous LGGs) were often thought to be virtually always *IDH* mutant lower grade gliomas [21]. Korber et al. evaluated a cohort of matched primary and recurrent *IDH* wild-type gliomas [53], and overall, contrary to the previous report [101], mutational burden was similar in primary and recurrent tumors, and 2/3 of recurrent tumors originated from multiple clones of the primary tumor (oligoclonal origin), while 1/3 were monoclonal. Additionally, although the heterogeneity in recurrent tumors was inherited from the primary lesion, most sub-clones in recurrent tumors showed additional driver mutations not found in the primary lesion, indicating ongoing genetic evolution [53]. CNVs in chromosomes 7, 9, and 10 were early alterations and preceded SNVs, similar to several previous studies in spatial heterogeneity [23,26,51,52]. Among the SNVs, *TERT* promoter, *TP53*, *PTEN* and *EGFR* mutations were the most frequent clonal alterations. *TERT* mutations exhibited a survival advantage over other early mutations, such as *CDKN2A/B* and *PTEN*. More importantly, through high-level mathematical estimates, the authors were able to propose that an *IDH* wild-type GBM may grow for 2–7 years undetected [53]. Thus, early diagnosis and surgical approaches may potentially identify a primary *IDH* wild-type LGG that evolves in a secondary GBM.

## 4. Future Directions

Over the next decade, the investigation of intra-tumor heterogeneity in GBM will likely increase in scope and detail. Proteomics and metabolomics are areas with opportunities for expanded understanding of gliomas. Nevertheless, inter- and intra-tumor differences in proteome profile have been reported in small series of adult patients [102,103,104], and in pediatric neuro-oncology [105]. Multi-institutional studies in adult populations are needed, aiming to identify molecular targets not observed from RNAseq data, as gene expression data often does not correlate highly to changes in proteomic expression. When paired with genomic sequencing, proteomic evaluation can better tease apart protein-level effects of gene mutations in GBMs, and help identify new IHC markers to be implemented for routine neuropathological testing. In addition, multiregional sampling of GBMs may discriminate functional differences between non-necrotic, peri-necrotic, and necrotic tissue, and potentially yield insights into mechanisms underlying treatment resistance related to changes in the TME. Batch effects in mass spectrometry (MS), and the lower quality of FFPE samples compared to fresh-frozen tissue is still an issue in proteomic studies, particularly in phospho-proteomic studies. We have demonstrated quality improvement in MS analysis of FFPE samples, which enabled the identification of differential proteomic profiles between *IDH* wild-type and *IDH* mutant gliomas, as well as among multiple areas of LGGs and GBMs, reflecting different molecular pathways [106,107]. Similar to other methods, it is expected that proteomics will advance for analyses at single-cell level.

The rapidly expanding field of metabolomics is another area of study where opportunities exist to better understand glioma biology. Recent advances, such as desorption electrospray ionization mass spectrometry (DESI-MS), can allow for finely detailed spatial resolution of lipids and metabolites from biological samples [108,109]. In coming years, the 2D slices of DESI-MS will likely be expanded in order to recreate the 3D metabolic structure of tumors through combination of individual 2D slices. This may better show how gliomas secure nutrients and other metabolites crucial for continued growth, which could be used for the development of new markers for functional image exams. Given the phenotypic differences given by *IDH* mutations in gliomas and its product 2-HG, which interferes in the Kreb’s Cycle, there is opportunity for exploration of the metabolic consequences of these mutations for GBMs.

The use of new methods to characterize tumor heterogeneity in GBM at protein and metabolite levels will fuel the next revolution in functional image examinations, which will trigger further improvements in early diagnosis, efficacy of surgical resection, histomolecular classification, and drug development, thereby substantially contributing to the advancement of personalized care for patients with GBM.

## Figures and Tables

**Table 1:** Summary of the histopathological, immunohistochemical, and molecular features of Grade 4 *IDH*-mutant gliomas and glioblastomas.

			Astrocytoma *IDH*mut WHO Grade 4	Glioblastoma *(IDH* Wild-Type)		References
		**Transcriptomic subtype**	**Proneural**	**Classical**	**Mesenchymal**	[18]
		**Histogenesis**	**Oligodendrocytes**	**Astrocytes**	**Astroglial cells**	[12,13,14]
		**Immunologic score**	**Cold**	**Cold**	**Hot**	[29,66,71,77]
**Histopathologic features**	**Traditional histologic features**	**Nuclear atypia/ pleomorphism**	Present	Present	Increased	[14,27]
		**Mitotic activity**	Present	Increased	Present	[14,27]
		**Microvascular proliferation**	Present	Increased	Present	[14,27]
		**Necrosis**	Present	Present	Extensive	[14,71]
		**Small cell component**	Not frequent	Increased	Frequent	[34,35]
		**Suggested IHC markers**	ASCL1, OLIG2, and PDGFRα	EGFR	p53, MET, ALDH1A3, IBA-1	[27,28,29]
	**Immune component**	**TAMs**	Not frequent	Frequent (enhanced DCs signature)	Increased (enhanced M1 signature)	[66]
		**TILs**	Not frequent (reduced CD4+ lymphocytes signature)	Frequent	Increased CD3+ and CD8+ lymphocytes	[66,77]
**Molecular features**	**WHO 2016 prognostic criteria**	***IDH* status**	Mutated	Wild type	Wild type	[15,16,17,18]
		***MGMT* promoter methylation**	More frequently methylated	More frequently unmethylated	More frequently unmethylated	[15,16,17,18]
		**G-CIMP**	Frequent	Rare	Absent	[16]
	**C-IMPACT-NOW 6 molecular criteria**	**Chromosome 7 gains (*EGFR*)**	Frequent	Present	Frequent	[11,26]
		**Chromosome 9 loss (*CDKN2A/B*)**	Present	Present	Present	[11,26]
		**Chromosome 10 loss**	N/A	Present	Present	[11,26]
		**TERT mutations**	N/A	Present	Present	[11,26]
		**Stemness**	Increased	Increased	Present	[55]

*IDH*—Isocitrate dehydrogenase. WHO—World Health Organization. IHC—immunohistochemistry.TAMs—Tumor-associated macrophages. DCs—Dendritic cells. TILs—Tumor-infiltrating lymphocytes. *MGMT*—O^6^-methylguanine-DNA methyltransferase. G-CIMP—CpG Island Methylator Phenotype.

## Data Availability

Not applicable.

## References

[1] Ostrom Q.T., Patil N., Cioffi G., Waite K., Kruchko C., Barnholtz-Sloan J.S. (2020). CBTRUS Statistical Report: Primary Brain and Other Central Nervous System Tumors Diagnosed in the United States in 2013–2017. Neuro. Oncol..

[2] Hegi M.E., Diserens A.-C., Gorlia T., Hamou M.-F., De Tribolet N., Weller M., Kros J.M., Hainfellner J.A., Mason W., Mariani L. (2005). MGMTGene Silencing and Benefit from Temozolomide in Glioblastoma. N. Engl. J. Med..

[3] Stupp R., Hegi M.E., Mason W.P., Bent M.J.V.D., Taphoorn M.J.B., Janzer R.C., Ludwin S.K., Allgeier A., Fisher B., Belanger K. (2009). Effects of radiotherapy with concomitant and adjuvant temozolomide versus radiotherapy alone on survival in glioblastoma in a randomised phase III study: 5-year analysis of the EORTC-NCIC trial. Lancet Oncol..

[4] Stupp R., Mason W.P., van den Bent M.J., Weller M., Fisher B., Taphoorn M.J.B., Belanger K., Brandes A.A., Marosi C., Bogdahn U. (2005). Radiotherapy plus Concomitant and Adjuvant Temozolomide for Glioblastoma. N. Engl. J. Med..

[5] Stupp R., Taillibert S., Kanner A., Read W., Steinberg D.M., Lhermitte B., Toms S., Idbaih A., Ahluwalia M.S., Fink K. (2017). Effect of Tumor-Treating Fields Plus Maintenance Temozolomide vs Maintenance Temozolomide Alone on Survival in Patients With Glioblastoma. JAMA.

[6] Hanahan D., Weinberg R.A. (2011). Hallmarks of Cancer: The Next Generation. Cell.

[7] McGranahan N., Swanton C. (2017). Clonal Heterogeneity and Tumor Evolution: Past, Present, and the Future. Cell.

[8] Alexandrov L.B., Nik-Zainal S., Wedge D.C., Aparicio S.A.J.R., Behjati S., Biankin A.V., Bignell G.R., Bolli N., Borg A., Børresen-Dale A.-L. (2013). Signatures of mutational processes in human cancer. Nature.

[9] Lawrence M.S., Stojanov P., Mermel C.H., Robinson J.T., Garraway L.A., Golub T.R., Meyerson M.L., Gabriel S.B., Lander E.S., Getz G. (2014). Discovery and saturation analysis of cancer genes across 21 tumour types. Nat. Cell Biol..

[10] R Core Team (2018). R: A Language and Environment for Statistical Computing.

[11] Louis D.N., Wesseling P., Aldape K., Brat D.J., Capper D., Cree I.A., Eberhart C., Figarella-Branger D., Fouladi M., Fuller G.N. (2020). cIMPACT-NOW update 6: New entity and diagnostic principle recommendations of the cIMPACT-Utrecht meeting on future CNS tumor classification and grading. Brain Pathol..

[12] Bailey O.T. (1985). Genesis of the Percival Bailey-Cushing Classification of Gliomas. Pediatr. Neurosurg..

[13] Zülch K.J. (1980). Principles of the new World Health Organization (WHO) classification of brain tumors. Neuroradiology.

[14] Daumas-Duport C., Scheithauer B., O’Fallon J., Kelly P. (1988). Grading of astrocytomas. A simple and reproducible method. Cancer.

[15] Ceccarelli M., Barthel F.P., Malta T.M., Sabedot T.S., Salama S.R., Murray B.A., Morozova O., Newton Y., Radenbaugh A., Pagnotta S.M. (2016). Molecular Profiling Reveals Biologically Discrete Subsets and Pathways of Progression in Diffuse Glioma. Cell.

[16] Noushmehr H., Weisenberger D.J., Diefes K., Phillips H.S., Pujara K., Berman B.P., Pan F., Pelloski C.E., Sulman E.P., Bhat K.P. (2010). Identification of a CpG Island Methylator Phenotype that Defines a Distinct Subgroup of Glioma. Cancer Cell.

[17] Phillips H.S., Kharbanda S., Chen R., Forrest W.F., Soriano R.H., Wu T.D., Misra A., Nigro J.M., Colman H., Soroceanu L. (2006). Molecular subclasses of high-grade glioma predict prognosis, delineate a pattern of disease progression, and resemble stages in neurogenesis. Cancer Cell.

[18] Verhaak R.G., Hoadley K.A., Purdom E., Wang V., Qi Y., Wilkerson M.D., Miller C.R., Ding L., Golub T., Mesirov J.P. (2010). Integrated Genomic Analysis Identifies Clinically Relevant Subtypes of Glioblastoma Characterized by Abnormalities in PDGFRA, IDH1, EGFR, and NF1. Cancer Cell.

[19] Brennan C.W., Verhaak R.G.W., McKenna A., Campos B., Noushmehr H., Salama S.R., Zheng S., Chakravarty D., Sanborn J.Z., Berman S.H. (2013). The Somatic Genomic Landscape of Glioblastoma. Cell.

[20] The Cancer Genome Atlas Research Network (2008). Comprehensive genomic characterization defines human glioblastoma genes and core pathways. Nat. Cell Biol..

[21] Louis D.N., Ohgaki H., Wiestler O.D., Cavenee W.K., Ellison D.W., Figarella-Branger D., Perry A., Reifenberger G., Deimling A.V., Louis D.N., Ohgaki H., Wiestler O.D., Cavenee W.K. (2016). WHO Classification of Tumors of the Central Nervous System.

[22] Parsons D.W., Jones S., Zhang X., Lin J.C.-H., Leary R.J., Angenendt P., Mankoo P., Carter H., Siu I.-M., Gallia G.L. (2008). An Integrated Genomic Analysis of Human Glioblastoma Multiforme. Science.

[23] Suzuki H., Aoki K., Chiba K., Sato Y., Shiozawa Y., Shiraishi Y., Shimamura T., Niida A., Motomura K., Ohka F. (2015). Mutational landscape and clonal architecture in grade II and III gliomas. Nat. Genet..

[24] Brat D.J., Aldape K., Colman H., Figrarella-Branger D., Fuller G.N., Giannini C., Holland E.C., Jenkins R.B., Kleinschmidt-DeMasters B., Komori T. (2020). cIMPACT-NOW update 5: Recommended grading criteria and terminologies for IDH-mutant astrocytomas. Acta Neuropathol..

[25] Ono T., Shirahata M., Louis D.N., Von Deimling A. (2018). Grading of Diffuse Astrocytic Gliomas: A Review of Studies Before and After the Advent of IDH Testing. Semin. Neurol..

[26] Stichel D., Ebrahimi A., Reuss D., Schrimpf D., Ono T., Shirahata M., Reifenberger G., Weller M., Hänggi D., Wick W. (2018). Distribution of EGFR amplification, combined chromosome 7 gain and chromosome 10 loss, and TERT promoter mutation in brain tumors and their potential for the reclassification of IDHwt astrocytoma to glioblastoma. Acta Neuropathol..

[27] Orzan F., Pagani F., Cominelli M., Triggiani L., Calza S., De Bacco F., Medicina D., Balzarini P., Panciani P.P., Liserre R. (2020). A simplified integrated molecular and immunohistochemistry-based algorithm allows high accuracy prediction of glioblastoma transcriptional subtypes. Lab. Investig..

[28] Conroy S., Kruyt F.A., Joseph J.V., Balasubramaniyan V., Bhat K.P., Wagemakers M., Enting R.H., Walenkamp A.M.E., Dunnen W.F.A.D. (2014). Subclassification of Newly Diagnosed Glioblastomas through an Immunohistochemical Approach. PLoS ONE.

[29] Liesche-Starnecker F., Mayer K., Kofler F., Baur S., Schmidt-Graf F., Kempter J., Prokop G., Pfarr N., Wei W., Gempt J. (2020). Immunohistochemically Characterized Intratumoral Heterogeneity Is a Prognostic Marker in Human Glioblastoma. Cancers.

[30] Paulus W., Peiffer J. (1989). Intratumoral histologic heterogeneity of gliomas. A quantitative study. Cancer.

[31] Jackson R.J., Fuller G.N., Abi-Said D., Lang F.F., Gokaslan Z.L., Shi W.M., Wildrick D.M., Sawaya R. (2001). Limitations of stereotactic biopsy in the initial management of gliomas. Neuro Oncol..

[32] Sallinen S.L., Sallinen P.K., Haapasalo H.K., Helin H.J., Helén P.T., Schraml P., Kallioniemi O.P., Kononen J. (2000). Identification of differentially expressed genes in human gliomas by DNA microarray and tissue chip techniques. Cancer Res..

[33] Burger P.C., Vollmer R.T. (1980). Histologic factors of prognostic significance in the glioblastoma multiforme. Cancer.

[34] Burger P.C., Dubois P.J., Schold S.C., Smith K.R., Odom G.L., Crafts D.C., Giangaspero F. (1983). Computerized tomographic and pathologic studies of the untreated, quiescent, and recurrent glioblastoma multiforme. J. Neurosurg..

[35] Giangaspero F., Burger P.C. (1983). Correlations between cytologic composition and biologic behavior in the glioblastoma multiforme. A postmortem study of 50 cases. Cancer.

[36] Hu L.S., Hawkins-Daarud A., Wang L., Li J., Swanson K.R. (2020). Imaging of intratumoral heterogeneity in high-grade glioma. Cancer Lett..

[37] Hu L.S., Ning S., Eschbacher J.M., Baxter L.C., Gaw N., Ranjbar S., Plasencia J., Dueck A.C., Peng S., Smith K.A. (2016). Radiogenomics to characterize regional genetic heterogeneity in glioblastoma. Neuro Oncol..

[38] Inano R., Oishi N., Kunieda T., Arakawa Y., Kikuchi T., Fukuyama H., Miyamoto S. (2016). Visualization of heterogeneity and regional grading of gliomas by multiple features using magnetic resonance-based clustered images. Sci. Rep..

[39] Bell E.H., Pugh S.L., McElroy J.P., Gilbert M.R., Mehta M., Klimowicz A.C., Magliocco A., Bredel M., Robe P., Grosu A.-L. (2017). Molecular-Based Recursive Partitioning Analysis Model for Glioblastoma in the Temozolomide Era: A Correlative Analysis Based on NRG Oncology RTOG 0525. JAMA Oncol..

[40] Capper D., Mittelbronn M., Meyermann R., Schittenhelm J. (2007). Pitfalls in the assessment of MGMT expression and in its correlation with survival in diffuse astrocytomas: Proposal of a feasible immunohistochemical approach. Acta Neuropathol..

[41] Dahlrot R.H., Dowsett J., Fosmark S., Malmström A., Henriksson R., Boldt H., De Stricker K., Sørensen M.D., Poulsen H.S., Lysiak M. (2017). Prognostic value of O-6-methylguanine-DNA methyltransferase (MGMT) protein expression in glioblastoma excluding nontumour cells from the analysis. Neuropathol. Appl. Neurobiol..

[42] Hegi M.E., Liu L., Herman J.G., Stupp R., Wick W., Weller M., Mehta M.P., Gilbert M.R. (2008). Correlation of O6-Methylguanine Methyltransferase (MGMT) Promoter Methylation With Clinical Outcomes in Glioblastoma and Clinical Strategies to Modulate MGMT Activity. J. Clin. Oncol..

[43] Lalezari S., Chou A.P., Tran A., Solis O.E., Khanlou N., Chen W., Li S., Carrillo J.A., Chowdhury R., Selfridge J. (2013). Combined analysis of O6-methylguanine-DNA methyltransferase protein expression and promoter methylation provides optimized prognostication of glioblastoma outcome. Neuro Oncol..

[44] Lee S., Reid H., Elder R.H., Thatcher N., Margison G. (1996). Inter- and intracellular heterogeneity of O6-alkylguanine-DNA alkyltransferase expression in human brain tumours: Possible significance in nitrosourea therapy. Carcinogenesis.

[45] Muñoz-Hidalgo L., San-Miguel T., Megías J., Monleón D., Navarro L., Roldán P., Cerdá-Nicolás M., López-Ginés C. (2020). Somatic copy number alterations are associated with EGFR amplification and shortened survival in patients with primary glioblastoma. Neoplasia.

[46] Strommer K., Hamou M.F., Diggelmann H., De Tribolet N. (1990). Cellular and tumoural heterogeneity of EGFR gene amplification in human malignant gliomas. Acta Neurochir..

[47] Purkait S., Miller C.A., Kumar A., Sharma V., Pathak P., Jha P., Sharma M.C., Suri V., Suri A., Sharma B.S. (2016). ATRX in Diffuse Gliomas With its Mosaic/Heterogeneous Expression in a Subset. Brain Pathol..

[48] Yung W.K., Shapiro J.R., Shapiro W.R. (1982). Heterogeneous chemosensitivities of subpopulations of human glioma cells in culture. Cancer Res..

[49] Milinkovic V., Bancovik J., Rakic M., Milosevic N., Stanković T., Joković M., Milošević Z., Skender-Gazibara M., Podolski-Renić A., Pešić M. (2012). Genomic instability and p53 alterations in patients with malignant glioma. Exp. Mol. Pathol..

[50] Stieber D., Golebiewska A., Evers L., Lenkiewicz E., Brons N.H.C., Nicot N., Oudin A., Bougnaud S., Hertel F., Bjerkvig R. (2013). Glioblastomas are composed of genetically divergent clones with distinct tumourigenic potential and variable stem cell-associated phenotypes. Acta Neuropathol..

[51] Coons S.W., Johnson P.C., Shapiro J.R. (1995). Cytogenetic and flow cytometry DNA analysis of regional heterogeneity in a low grade human glioma. Cancer Res..

[52] Harada K., Nishizaki T., Ozaki S., Kubota H., Ito H., Sasaki K. (1998). Intratumoral cytogenetic heterogeneity detected by comparative genomic hybridization and laser scanning cytometry in human gliomas. Cancer Res..

[53] Körber V., Yang J., Barah P., Wu Y., Stichel D., Gu Z., Fletcher M.N.C., Jones D., Hentschel B., Lamszus K. (2019). Evolutionary Trajectories of IDHWT Glioblastomas Reveal a Common Path of Early Tumorigenesis Instigated Years ahead of Initial Diagnosis. Cancer Cell.

[54] Soda Y., Marumoto T., Friedmann-Morvinski D., Soda M., Liu F., Michiue H., Pastorino S., Yang M., Hoffman R.M., Kesari S. (2011). Transdifferentiation of glioblastoma cells into vascular endothelial cells. Proc. Natl. Acad. Sci. USA.

[55] Jung V., Romeike B.F.M., Henn W., Feiden W., Moringlane J.R., Zang K.D., Urbschat S. (1999). Evidence of Focal Genetic Microheterogeneity in Glioblastoma Multiforme by Area-Specific CGH on Microdissected Tumor Cells. J. Neuropathol. Exp. Neurol..

[56] Patel A.P., Tirosh I., Trombetta J.J., Shalek A.K., Gillespie S.M., Wakimoto H., Cahill D.P., Nahed B.V., Curry W.T., Martuza R.L. (2014). Single-cell RNA-seq highlights intratumoral heterogeneity in primary glioblastoma. Science.

[57] Little S.E., Popov S., Jury A., Bax D.A., Doey L., Al-Sarraj S., Jurgensmeier J.M., Jones C. (2012). Receptor Tyrosine Kinase Genes Amplified in Glioblastoma Exhibit a Mutual Exclusivity in Variable Proportions Reflective of Individual Tumor Heterogeneity. Cancer Res..

[58] Snuderl M., Fazlollahi L., Le L.P., Nitta M., Zhelyazkova B.H., Davidson C.J., Akhavanfard S., Cahill D.P., Aldape K.D., Betensky R.A. (2011). Mosaic Amplification of Multiple Receptor Tyrosine Kinase Genes in Glioblastoma. Cancer Cell.

[59] Decarvalho A., Kim H., Poisson L.M., Winn M.E., Mueller C., Cherba D., Koeman J., Seth S., Protopopov A., Felicella M. (2018). Discordant inheritance of chromosomal and extrachromosomal DNA elements contributes to dynamic disease evolution in glioblastoma. Nat. Genet..

[60] Hoffman L.M., DeWire M., Ryall S., Buczkowicz P., Leach J.L., Miles L., Ramani A., Brudno M., Kumar S.S., Drissi R. (2016). Spatial genomic heterogeneity in diffuse intrinsic pontine and midline high-grade glioma: Implications for diagnostic biopsy and targeted therapeutics. Acta Neuropathol. Commun..

[61] Vinci M., Burford A., Molinari V., Kessler K., Popov S., Clarke M., Taylor K.R., Pemberton H.N., Lord C.J., Gutteridge A. (2018). Functional diversity and cooperativity between subclonal populations of pediatric glioblastoma and diffuse intrinsic pontine glioma cells. Nat. Med..

[62] Akgül S., Patch A.-M., D’Souza R.C.J., Mukhopadhyay P., Nones K., Kempe S., Kazakoff S., Jeffree R., Stringer B.W., Pearson J.V. (2019). Intratumoural Heterogeneity Underlies Distinct Therapy Responses and Treatment Resistance in Glioblastoma. Cancers.

[63] Feng L., Qian H., Yu X., Liu K., Xiao T., Zhang C., Kuang M., Cheng S., Li X., Wan J. (2017). Heterogeneity of tumor-infiltrating lymphocytes ascribed to local immune status rather than neoantigens by multi-omics analysis of glioblastoma multiforme. Sci. Rep..

[64] Gates E., Yang J., Fukumura K., Lin J.S., Weinberg J.S., Prabhu S.S., Long L., Fuentes D., Sulman E.P., Huse J.T. (2019). Spatial Distance Correlates With Genetic Distance in Diffuse Glioma. Front. Oncol..

[65] Lee J.-K., Wang J., Sa J.K., Ladewig E., Lee H.-O., Lee I.-H., Kang H.J., Rosenbloom D.S., Camara P.G., Liu Z. (2017). Spatiotemporal genomic architecture informs precision oncology in glioblastoma. Nat. Genet..

[66] Mohme M., Maire C.L., Riecken K., Zapf S., Aranyossy T., Westphal M., Lamszus K., Fehse B. (2017). Optical Barcoding for Single-Clone Tracking to Study Tumor Heterogeneity. Mol. Ther..

[67] Wang Q., Hu B., Hu X., Kim H., Squatrito M., Scarpace L., Decarvalho A.C., Lyu S., Li P., Li Y. (2017). Tumor Evolution of Glioma-Intrinsic Gene Expression Subtypes Associates with Immunological Changes in the Microenvironment. Cancer Cell.

[68] Sottoriva A., Spiteri I., Piccirillo S.G.M., Touloumis A., Collins V.P., Marioni J.C., Curtis C., Watts C., Tavaré S. (2013). Intratumor heterogeneity in human glioblastoma reflects cancer evolutionary dynamics. Proc. Natl. Acad. Sci. USA.

[69] Mackay A., Burford A., Carvalho D., Izquierdo E., Fazal-Salom J., Taylor K.R., Bjerke L., Clarke M., Vinci M., Nandhabalan M. (2017). Integrated Molecular Meta-Analysis of 1,000 Pediatric High-Grade and Diffuse Intrinsic Pontine Glioma. Cancer Cell.

[70] Bonavia R., Inda M.-D.-M., Cavenee W.K., Furnari F.B. (2011). Heterogeneity Maintenance in Glioblastoma: A Social Network. Cancer Res..

[71] Meyer M., Reimand J., Lan X., Head R., Zhu X., Kushida M., Bayani J., Pressey J.C., Lionel A.C., Clarke I.D. (2015). Single cell-derived clonal analysis of human glioblastoma links functional and genomic heterogeneity. Proc. Natl. Acad. Sci. USA.

[72] Klughammer J., Kiesel B., Roetzer T., Fortelny N., Nemc A., Nenning K.-H., Furtner J., Sheffield N.C., Datlinger P., Peter N. (2018). The DNA methylation landscape of glioblastoma disease progression shows extensive heterogeneity in time and space. Nat. Med..

[73] Mazor T., Pankov A., Johnson B.E., Hong C., Hamilton E.G., Bell R.J., Smirnov I.V., Reis G.F., Phillips J.J., Barnes M.J. (2015). DNA Methylation and Somatic Mutations Converge on the Cell Cycle and Define Similar Evolutionary Histories in Brain Tumors. Cancer Cell.

[74] Abou-El-Ardat K., Seifert M., Becker K., Eisenreich S., Lehmann M., Hackmann K., Rump A., Meijer G., Carvalho B., Temme A. (2017). Comprehensive molecular characterization of multifocal glioblastoma proves its monoclonal origin and reveals novel insights into clonal evolution and heterogeneity of glioblastomas. Neuro Oncol..

[75] Hayes J., Yu Y., Jalbert L.E., Mazor T., Jones L.E., Wood M.D., Walsh K.M., Bengtsson H., Hong C., Oberndorfer S. (2017). Genomic analysis of the origins and evolution of multicentric diffuse lower-grade gliomas. Neuro Oncol..

[76] Liu Q., Liu Y., Li W., Wang X., Sawaya R., Lang F.F., Yung W.K.A., Chen K., Fuller G.N., Zhang W. (2015). Genetic, epigenetic, and molecular landscapes of multifocal and multicentric glioblastoma. Acta Neuropathol..

[77] Dabrowski M.J., Draminski M., Diamanti K., Stepniak K., Mozolewska M.A., Teisseyre P., Koronacki J., Komorowski J., Kaminska B., Wojtas B. (2018). Unveiling new interdependencies between significant DNA methylation sites, gene expression profiles and glioma patients survival. Sci. Rep..

[78] Dejaegher J., Solie L., Hunin Z., Sciot R., Capper D., Siewert C., Van Cauter S., Wilms G., Van Loon J., Ectors N. (2020). OUP accepted manuscript. Neuro Oncol..

[79] Feng Y., Li G., Shi Z., Yan X., Wang Z., Jiang H., Chen Y., Li R., Zhai Y., Chang Y. (2020). A novel methylation signature predicts radiotherapy sensitivity in glioma. Sci. Rep..

[80] Valor L.M., Hervás-Corpión I. (2020). The Epigenetics of Glioma Stem Cells: A Brief Overview. Front. Oncol..

[81] Wang Z., Gao L., Guo X., Lian W., Deng K., Xing B. (2020). Development and Validation of a Novel DNA Methylation-Driven Gene Based Molecular Classification and Predictive Model for Overall Survival and Immunotherapy Response in Patients With Glioblastoma: A Multiomic Analysis. Front. Cell Dev. Biol..

[82] Pangeni R., Zhang Z., Alvarez A.A., Wan X., Sastry N., Lu S., Shi T., Huang T., Lei C.X., James C.D. (2018). Genome-wide methylomic and transcriptomic analyses identify subtype-specific epigenetic signatures commonly dysregulated in glioma stem cells and glioblastoma. Epigenetics.

[83] Domingues P., González-Tablas M., Otero Á., Pascual D., Miranda D., Ruiz L., Sousa P., Ciudad J., Gonçalves J.M., Lopes M.C. (2016). Tumor infiltrating immune cells in gliomas and meningiomas. Brain Behav. Immun..

[84] Hussain S.F., Yang D., Suki D., Aldape K., Grimm E., Heimberger A.B. (2006). The role of human glioma-infiltrating microglia/macrophages in mediating antitumor immune responses. Neuro Oncol..

[85] Stenken J.A., Poschenrieder A.J. (2015). Bioanalytical chemistry of cytokines–A review. Anal. Chim. Acta.

[86] Morantz R.A., Wood G.W., Foster M., Clark M., Gollahon K. (1979). Macrophages in experimental and human brain tumors: Part 2: Studies of the macrophage content of human brain tumors. J. Neurosurg..

[87] Phillips J.P., Eremin O., Anderson J.R. (1982). Lymphoreticular cells in human brain tumours and in normal brain. Br. J. Cancer.

[88] Wood G.W., Morantz R.A. (1979). Immunohistologic Evaluation of the Lymphoreticular Infiltrate of Human Central Nervous System Tumors. J. Natl. Cancer Inst..

[89] Hambardzumyan D., Gutmann D.H., Kettenmann H. (2016). The role of microglia and macrophages in glioma maintenance and progression. Nat. Neurosci..

[90] Mantovani A., Sozzani S., Locati M., Allavena P., Sica A. (2002). Macrophage polarization: Tumor-associated macrophages as a paradigm for polarized M2 mononuclear phagocytes. Trends Immunol..

[91] Galon J., Bruni D. (2019). Approaches to treat immune hot, altered and cold tumours with combination immunotherapies. Nat. Rev. Drug Discov..

[92] Galon J., Costes A., Sanchez-Cabo F., Kirilovsky A., Mlecnik B., Lagorce-Pagès C., Tosolini M., Camus M., Berger A., Wind P. (2006). Type, Density, and Location of Immune Cells Within Human Colorectal Tumors Predict Clinical Outcome. Science.

[93] Tomaszewski W., Sanchez-Perez L., Gajewski T.F., Sampson J.H. (2019). Brain Tumor Microenvironment and Host State: Implications for Immunotherapy. Clin. Cancer Res..

[94] Bunse L., Pusch S., Bunse T., Sahm F., Sanghvi K., Friedrich M., AlAnsary D., Sonner J.K., Green E., Deumelandt K. (2018). Suppression of antitumor T cell immunity by the oncometabolite (R)-2-hydroxyglutarate. Nat. Med..

[95] Schalper K.A., Rodriguez-Ruiz M.E., Diez-Valle R., López-Janeiro A., Porciuncula A., Idoate M.A., Inogés S., De Andrea C., De Cerio A.L.-D., Tejada S. (2019). Neoadjuvant nivolumab modifies the tumor immune microenvironment in resectable glioblastoma. Nat. Med..

[96] Cloughesy T.F., Mochizuki A.Y., Orpilla J.R., Hugo W., Lee A.H., Davidson T.B., Wang A.C., Ellingson B.M., Rytlewski J.A., Sanders C.M. (2019). Neoadjuvant anti-PD-1 immunotherapy promotes a survival benefit with intratumoral and systemic immune responses in recurrent glioblastoma. Nat. Med..

[97] Bockmayr M., Klauschen F., Maire C.L., Rutkowski S., Westphal M., Lamszus K., Schüller U., Mohme M. (2019). Immunologic Profiling of Mutational and Transcriptional Subgroups in Pediatric and Adult High-Grade Gliomas. Cancer Immunol. Res..

[98] Zhai L., Ladomersky E., Lauing K.L., Wu M., Genet M., Gritsina G., Győrffy B., Brastianos P.K., Binder D.C., Sosman J.A. (2017). Infiltrating T Cells Increase IDO1 Expression in Glioblastoma and Contribute to Decreased Patient Survival. Clin. Cancer Res..

[99] Sanai N., Berger M.S. (2018). Surgical oncology for gliomas: The state of the art. Nat. Rev. Clin. Oncol..

[100] Zhao Y.-H., Wang Z.-F., Pan Z.-Y., Péus D., Delgado-Fernandez J., Pallud J., Li Z.-Q. (2019). A Meta-Analysis of Survival Outcomes Following Reoperation in Recurrent Glioblastoma: Time to Consider the Timing of Reoperation. Front. Neurol..

[101] Barthel F.P., Johnson K.C., Varn F.S., Moskalik A.D., Tanner G., Kocakavuk E., Anderson K.J., Abiola O., Aldape K., Alfaro K.D. (2019). Longitudinal molecular trajectories of diffuse glioma in adults. Nature.

[102] De Aquino P.F., Carvalho P.C., Nogueira F.C.S., Da Fonseca C.O., Silva J.C.T.D.S., Carvalho M.D.G.D.C., Domont G.B., Zanchin N.I.T., Fischer J.D.S.D.G. (2016). A Time-Based and Intratumoral Proteomic Assessment of a Recurrent Glioblastoma Multiforme. Front. Oncol..

[103] Fang X., Wang C., Balgley B.M., Zhao K., Wang W., He F., Weil R.J., Lee C.S. (2012). Targeted Tissue Proteomic Analysis of Human Astrocytomas. J. Proteome Res..

[104] Park C.-K., Jung J.H., Park S.-H., Jung H.-W., Cho B.-K. (2009). Multifarious proteomic signatures and regional heterogeneity in glioblastomas. J. Neuro Oncol..

[105] Petralia F., Tignor N., Reva B., Koptyra M., Chowdhury S., Rykunov D., Krek A., Ma W., Zhu Y., Ji J. (2020). Integrated Proteogenomic Characterization across Major Histological Types of Pediatric Brain Cancer. Cell.

[106] Becker A.P., Bell E.H., Haque S.J., McElroy J., Fleming J., Han C., Popp I., Prinz M., Straszewski O., Grosu A. (2019). Path-19. Tumor heterogeneity in gliomas: A pilot study of histopathology-associated proteome profiles assessed by liquid chromatography tandem mass spectrometry of ffpe samples. Neuro Oncol..

[107] Becker A.P., Bell E.H., Haque S.J., McElroy J., Prinz M., Staszewski O., Han C., Fleming J., Popp I., Grosu A. (2019). Tumor heterogeneity in gliomas–A histopathology-targeted proteomic pilot study. Int. J. Radiat. Oncol. Biol. Phys..

[108] Ait-Belkacem R., Berenguer C., Villard C., Ouafik L., Figarella-Branger M., Chinot O.L., Lafitte D. (2013). MALDI imaging and in-source decay for top-down characterization of glioblastoma. Proteomics.

[109] Eberlin L.S. (2014). DESI-MS Imaging of Lipids and Metabolites from Biological Samples. Mass Spectrom. Metab..

